# MMP7 Shedding of Syndecan-1 Facilitates Re-Epithelialization by Affecting α_2_β_1_ Integrin Activation

**DOI:** 10.1371/journal.pone.0006565

**Published:** 2009-08-10

**Authors:** Peter Chen, Laura E. Abacherli, Samuel T. Nadler, Ying Wang, Qinglang Li, William C. Parks

**Affiliations:** Center for Lung Biology, Department of Medicine, University of Washington, Seattle, Washington, United States of America; Johns Hopkins School of Medicine, United States of America

## Abstract

**Background:**

Lung injury promotes the expression of matrix metalloproteinase-7 (MMP7, matrilysin), which is required for neutrophil recruitment and re-epithelialization. MMP7 governs the lung inflammatory response through the shedding of syndecan-1. Because inflammation and repair are related events, we evaluated the role of syndecan-1 shedding in lung re-epithelialization.

**Methodology/Principal Finding:**

Epithelial injury induced syndecan-1 shedding from wild-type epithelium but not from *Mmp7^−/−^* mice *in vitro* and *in vivo*. Moreover, cell migration and wound closure was enhanced by MMP7 shedding of syndecan-1. Additionally, we found that syndecan-1 augmented cell adhesion to collagen by controlling the affinity state of the α_2_β_1_ integrin.

**Conclusion/Significance:**

MMP7 shedding of syndecan-1 facilitates wound closure by causing the α_2_β_1_ integrin to assume a less active conformation thereby removing restrictions to migration. MMP7 acts in the lungs to regulate inflammation and repair, and our data now show that both these functions are controlled through the shedding of syndecan-1.

## Introduction

Being contiguous with the environment, mucosal surfaces are constantly exposed to toxic and pathogenic insults [1], [2]. As a first line of defense, mucosal epithelia have evolved to quickly respond to various forms of injury by coordinating the inflammatory response while repairing wounded tissue. MMP7 (matrilysin), a member of the matrix metalloproteinase (MMP) family, is expressed by all mucosal surfaces and is quickly upregulated in response to epithelial injury [3]–[11]. Accordingly, MMP7 functions to facilitate repair and regulate the acute inflammatory response [3]–[7].

The lungs are lined by a prototypical mucosal epithelium. Like other mucosal surfaces, injured lungs quickly initiate a pre-programmed response to recruit inflammatory cells and repair the damaged tissue. The roles of MMP7 in regulating repair and inflammation are particularly prominent in the lungs. In fact, the lung phenotypes are so pronounced that re-epithelialization and neutrophil recruitment into the alveolar space are almost completely abrogated in MMP7-deficient mice [3]–[6]. Our group previously reported that E-cadherin is shed *in vivo* from the injured lung epithelium by MMP7 [5]. Although we then proposed that E-cadherin shedding could promote repair by reorganizing cell-cell contacts, newer studies indicate that MMP7 shedding of E-cadherin functions in adaptive immune responses later in the response to injury (McGuire et al., unpublished observations). Early in the injury response, MMP7 promotes inflammation by shedding syndecan-1/KC (CXCL8) complexes that permit the transepithelial movement of neutrophils [4]. As shedding of syndecan-1 occurs coincident with the onset of re-epithelialization, we hypothesized that proteolysis of this membrane factor may also function in repair.

Syndecan-1 is one of four members of a family of transmembrane heparan sulfate proteoglycans with distinct expression patterns and functions [12]. Epithelial cells primarily express syndecan-1, and observations from various models indicate that it participates in wound healing [4], [13]–[17]. For example, suppression of syndecan-1 expression in epithelial cells induces a pro-migratory phenotype [18], [19], suggesting that intact syndecan-1 may moderate re-epithelialization. Consistent with this idea, syndecan-1 surface levels are decreased in injured cornea and skin during active repair [20], [21], and increased levels of syndecan-1 ectodomain are present in dermal wound fluid [22]–[24]. Syndecan-1 shedding from the cell surface is a MMP-dependent process *in vitro* and *in vivo*, and MMP shedding of syndecan-1 induces cell migration [25]. Moreover, MMP7 has been identified as the syndecan-1 sheddase in lung mucosa [4], [25]–[29].

Because it can modulate repair, we tested the idea that shedding of syndecan-1 from injured lung epithelium functions to promote re-epithelialization. Using *in vitro* and *in vivo* models, we found that syndecan-1 is shed from repairing epithelial cells after injury. Additionally, MMP7 shedding of syndecan-1 enhances cell migration and wound closure. Our data further demonstrates that syndecan-1 restrains cell migration by modifying the activation state of the α_2_β_1_ integrin. Our results establish that MMP7 shedding of syndecan-1 facilitates lung re-epithelialization and acts as a unified mechanism that regulates both acute inflammation and repair.

## Results

### MMP7 is required for cell migration

Air-liquid interface (ALI) cultures of airway epithelial cells differentiate into a complete mucociliary epithelium and act phenotypically similar to the *in vivo* epithelium thus providing a relevant organotypic culture system to study the airway mucosal epithelium [6]. We wounded wild-type (WT) and MMP7-null (*Mmp7^−/−^*) ALI cultures and observed the wound closure with time-lapse microscopy (Video S1). Whereas injured WT epithelium covered the wound in the field of view within 24 h, *Mmp7^−/−^* cells had a dramatic inability to close the wound. Wounded epithelium responds to injury by initially spreading over the wound followed by cell proliferation and migration over the damaged areas [30], [31]. Time-lapse microscopy revealed that the *Mmp7^−/−^* epithelium appeared to retain the ability to spread over the wound, as these cells formed extended lamellipodial fronts soon after wounding. However, only WT epithelium continued to migrate and complete the wound healing process. These data confirm that MMP7 is required for re-epithelialization.

### MMP7 shedding of syndecan-1 in repair

**Figure 1:**
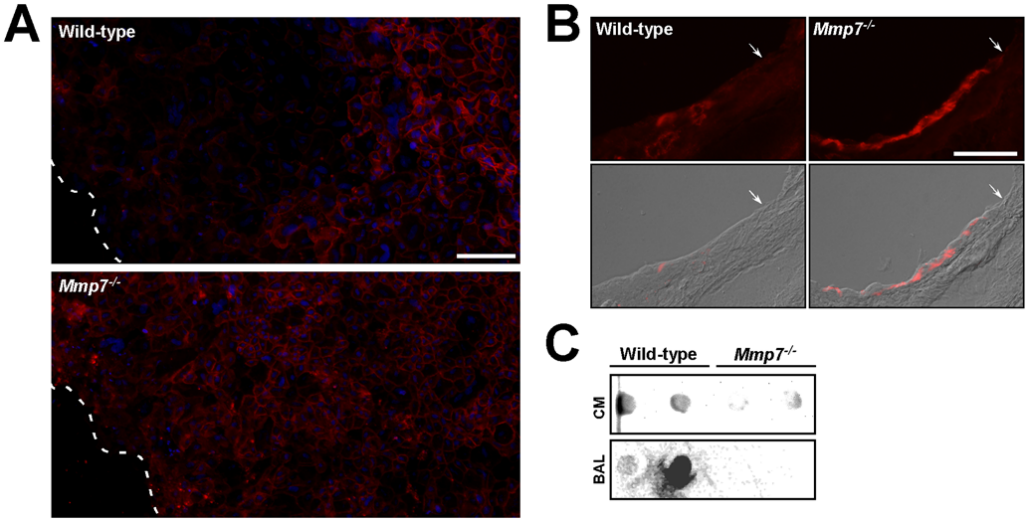
Syndecan-1 shedding from injured lung epithelium. (A) ALI cultures 24 h after wounding and (B) lungs two days after naphthalene injury were processed for syndecan-1 immunostaining (scale bar = 100 µm). ALI culture sections were counterstained with Dapi (blue). The white dashed line and the white arrows demarcate the wound front in ALI cultures and naphthalene-injured airway epithelium, respectively. Images are representative of consistent findings in several replicates (n≥3 ALI cultures or mice). (C) Syndecan-1 dot blot was performed using the 281-2 antibody (1∶1000) as previously described [4] on conditioned medium (CM) from injured ALI cultures and from bronchoalveolar lavage (BAL) fluid collected from lungs four days after naphthalene injury. Two independent samples were blotted from each genotype, but the leftmost WT BAL sample did not completely flow.

Because MMP7 sheds syndecan-1 from lung epithelium in response to injury [4], we evaluated if release of this proteoglycan functions in re-epithelialization. Immunofluorescence signal for syndecan-1 was decreased at the wound front of WT ALI cultures but remained in *Mmp7^−/−^* epithelium after injury (Figure 1A). Cells distal to the wound, representing uninjured epithelium, had equivalent syndecan-1 signal between WT and *Mmp7^−/−^* cultures. We also evaluated re-epithelialization *in vivo* using the naphthalene injury model. Naphthalene specifically kills Clara cells, which make up about 60% of airway epithelium in mice, while sparing other epithelial cell types and with minimal inflammation [6], [32]. In a well-described pattern of repair, the remaining epithelium becomes squamated and migrates to cover the denuded areas left by the sloughed Clara cells. By 14 days post-injury, the epithelium is repaired, and the Clara cell population is fully restored. Using this model, we observed syndecan-1 signal persisted in the *Mmp7^−/−^* epithelium after injury but was diminished in WT airway epithelium (Figure 1B). Vehicle-injected WT and *Mmp7^−/−^* mice had similar levels of syndecan-1 signal in an expected basolateral distribution (data not shown). Moreover, shed syndecan-1 was detected in the medium of injured WT cultures and in bronchoalveolar lavage fluid from naphthalene injured WT mice but not in *Mmp7^−/−^* samples (Figure 1C). These *in vitro* and *in vivo* findings confirm that MMP7 sheds syndecan-1 from injured lung epithelial cells.

### MMP7 releases syndecan-1 restrictions on wound closure

**Figure 2:**
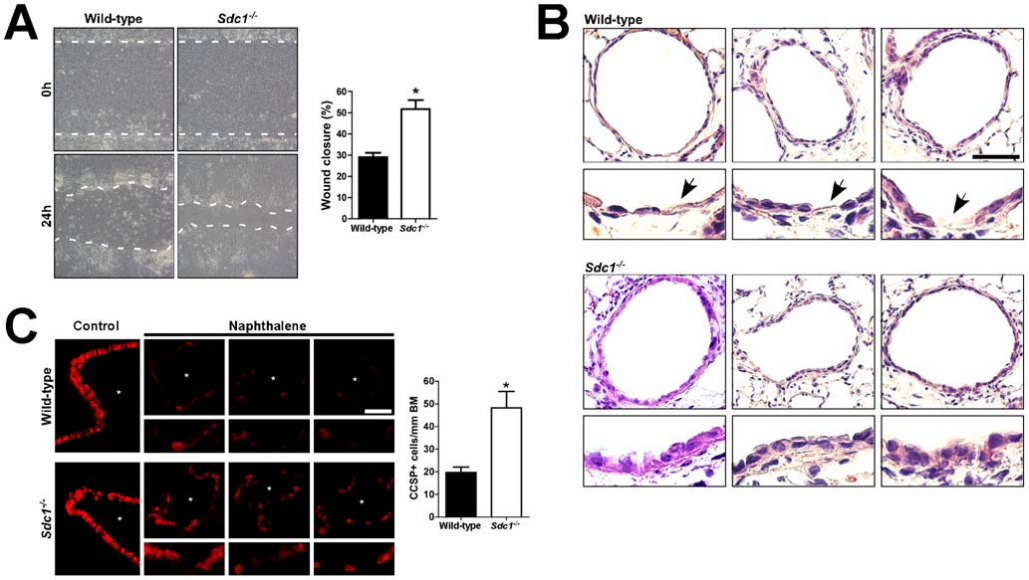
Syndecan-1 restrains lung re-epithelialization. (A) Wild-type and *Sdc1^−/−^* ALI cultures were wounded, and the repair was quantified. Each experiment was performed in triplicate and repeated at least 6 times. Original magnification×100. *p<0.0005 by Student's T-Test. (B) Wild-type and *Sdc1^−/−^* mice 4 days after naphthalene injury were processed for (B) H&E staining and (C) CCSP immunostaining (scale bar = 100 µm). Each panel is from a different mouse and has an accompanying enlarged portion of the airway (i.e., airways from 3 different mice were shown). In H&E stained sections, *Sdc1^−/−^* airway epithelium was more cuboidal in appearance. In contrast, wild-type epithelium was predominantly squamous with persistently exposed substratum (arrows). Additionally, the number of CCSP+cells per linear length of basement membrane (BM) along the airways (asterisk) was determined to quantify the epithelial repair after naphthalene injury. n = 4 mice, *p<0.01 by Student's T-Test.

The observations that migration and syndecan-1 shedding were diminished in *Mmp7^−/−^* tissue and cells after injury suggested that release of syndecan-1 is needed to promote re-epithelialization. To study this idea, we injured syndecan-1 null (*Sdc1^−/−^*) ALI cultures, which grew and differentiated indistinguishable from WT cultures, and found wounds closed significantly faster than in WT cultures (Figure 2A). Additionally, following naphthalene injury, re-epithelialization *in vivo* was quantitatively faster in *Sdc1^−/−^* mice with cuboidal cells appearing sooner compared to WT airways, in which the lining remained patchy and squamated at this time (Figure 2B). To quantify repair *in vivo*, we immunostained for Clara-cell specific protein (CCSP) and found the number of CCSP-positive cells along the airways was 2.5 times greater in *Sdc1^−/−^* mice at 4 days post-naphthalene compared to WT mice (Figure 2C). The airway epithelium in WT and *Sdc1^−/−^* mice was equivalent in vehicle-injected controls and had similar degrees of injury after naphthalene injury (data not shown). The accelerated wound closure in *Sdc1^−/−^* cultures and airways indicate that MMP7 shedding of syndecan-1 releases restrictions to epithelial cell movement.

**Figure 3:**
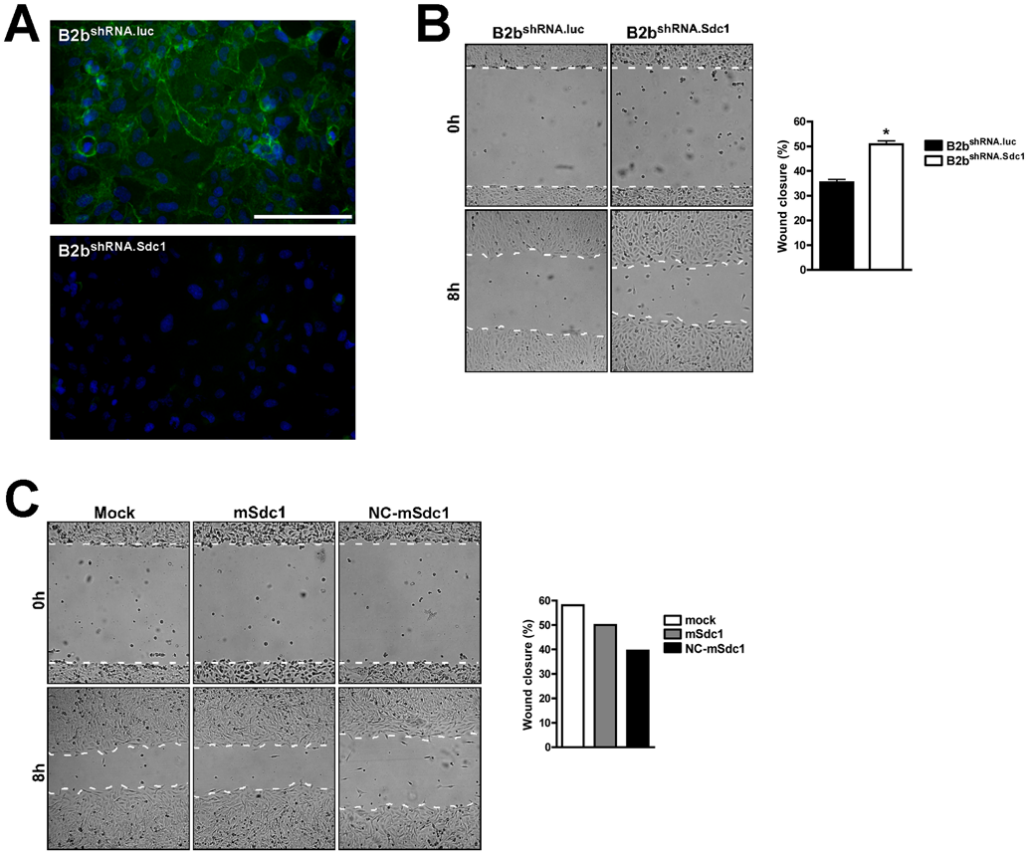
MMP7 shedding of syndecan-1 releases restrictions to migration. BEAS-2b cells were used to create stable knockdown cell lines using shRNA targeting either the luciferase gene as a control (B2b^shRNA.luc^) or syndecan-1 (B2b^shRNA.Sdc1^). Monolayers of B2b^shRNA.luc^ and B2b^shRNA.Sdc1^ cells were (A) immunostained for syndecan-1 (green; scale bar = 100 µm), and (B) scratched and wound closure was quantified. n = 4, p<0.0001 by Student's T-Test. (C) Wound closure was determined for B2b^shRNA.Sdc1^ cells stably overexpressing MMP7 and transiently transfected with no plasmid (Mock), mouse syndecan-1 (mSdc1) or a MMP7-resistant syndecan-1 mutant (NC-Sdc1). The graph is a representative figure of reproducible results on Type I collagen.

To understand better the mechanisms by which syndecan-1 restrains repair, we used a retroviral vector to create BEAS-2b cells (immortalized human bronchial airway epithelial cell line) that stably expressed shRNA that complements either human syndecan-1 mRNA (B2b^shRNA.Sdc1^) or a nonsense (luciferase) mRNA (B2b^shRNA.luc^). Syndecan-1 expression was markedly knocked down in B2b^shRNA.Sdc1^ cells but not altered in B2b^shRNA.luc^ cells, which had the expected basolateral distribution of the proteoglycan (Figure 3A). Cell monolayers were injured and revealed significantly faster wound closure in B2b^shRNA.Sdc1^ compared to B2b^shRNA.luc^ cells (Figure 3B). These findings recapitulated the more efficient re-epithelialization phenotype in *Sdc1^−/−^* ALI and naphthalene injury models and further support our conclusion that intact syndecan-1 functions to restrain migration.

**Figure 4:**
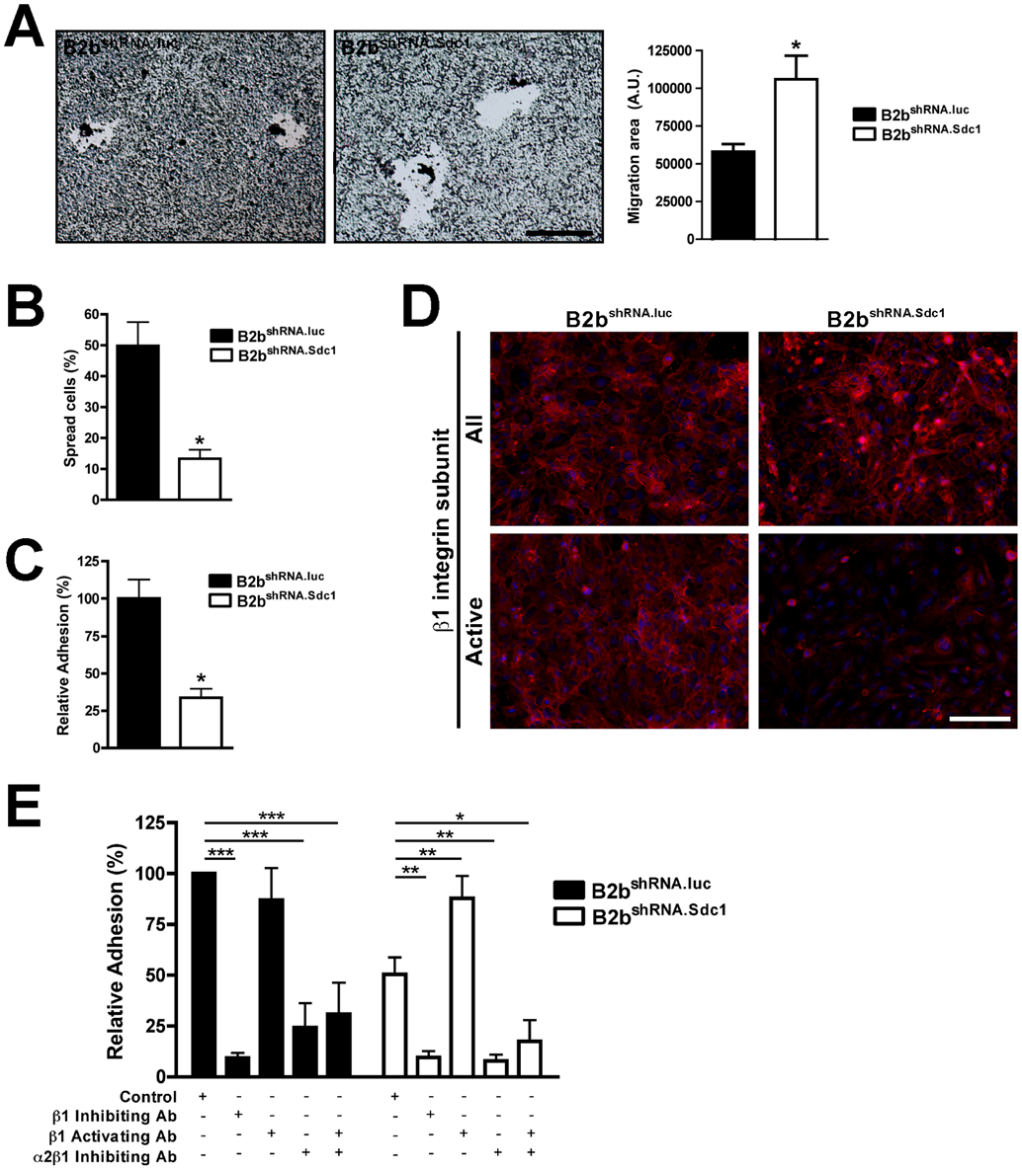
Syndecan-1 regulation of cell-matrix interactions. (A) B2b^shRNA.luc^ and B2b^shRNA.Sdc1^ cells were used in a gold colloid migration assay (scale bar = 100 µm). Total migration area was measured for cells plated on type I collagen. n = 4, *p<0.05 by Student's T-Test. (B) The percent of spread cells versus all cells was measured after plating on type I collagen. n = 5, *p<0.005 by Student's T-Test. (C) The relative adhesion percent for cells on type I collagen was determined. n = 6, *p<0.0005 by Student's T-Test. (D) Monolayers of B2b^shRNA.luc^ and B2b^shRNA.Sdc1^ cells were immunostained for the β_1_ integrin subunit (red) using all (clone AIIB2) or active conformation-specific (clone 12G10) antibodies. Immunofluorescent images counterstained with Dapi (scale bar = 100 µm). (E) The relative adhesion percent for cells on type I collagen was measured in the presence of control, β_1_ subunit inhibiting antibody (clone AIIB2; 1 µg/ml), β_1_ subunit activating antibody (clone HUTS-21; 10 µg/ml) and/or α_2_β_1_ integrin inhibiting antibody (clone BHA2.1, 20 µg/ml). Isotype control antibodies were matched to specific antibody experiment. n≥3, *p<0.05, **p<0.01, ***p<0.001 by 2-way ANOVA and Bonferroni analysis.

Because MMP7 was undetectable by western blot in conditioned medium from BEAS-2b cells (data not shown), faster migration seen with targeted ablation of syndecan-1 suggest that release of this surface proteoglycan–and not other potential substrates–is the MMP7-mediated mechanism that promotes re-epithelialization. We stably overexpressed active MMP7 in B2b^shRNA.Sdc1^ cells and transiently transfected these cells with constructs that express either wild-type mouse syndecan-1 (mSdc1), which would not be affected by the shRNA targeting the human transcript, or a mutant mouse syndecan-1 resistant to MMP7 proteolysis (NC-mSdc1). Cells expressing NC-mSdc1 had reduced rates of wound closure compared to cells expressing cleavable WT syndecan-1 (Figure 3C). To assess the role of syndecan-1 in cell migration directly, we used a phagokinetic colloid gold migration assay [33] to measure the movement of individual cells over 24 h (Figure 4A). Consistent with rapid repair seen in *Sdc1^−/−^* ALI cultures and tissue, cells that lack syndecan-1 (B2b^shRNA.Sdc1^) migrated faster than control cells (B2b^shRNA.luc^) (Figure 4A). Together, these findings indicate that MMP7 cleavage of syndecan-1 releases restrictions to migration.

### Syndecan-1 affects cell adhesion and spreading

The ability of a cell to adhere and spread over a matrix substratum is essential for migration. Although they migrated faster, cell spreading was significantly blunted in B2b^shRNA.Sdc1^ cells compared to B2b^shRNA.luc^ cells (13.3%±2.9 versus 49.8%±7.7, respectively; p<0.005; Figure 4B). Furthermore, B2b^shRNA.Sdc1^ cells were two-fold less adherent to type I collagen compared to B2b^shRNA.luc^ cells after 30 min of contact (p<0.0001; Figure 4C). However, knock-down of syndecan-1 did not completely ablate the ability to ligate collagen and only changed the kinetics of adhesion as both B2b^shRNA.Sdc1^ and B2b^shRNA.luc^ cell were attached to the substratum by 4 h (data not shown).

### Syndecan-1 regulates α_2_β_1_ integrin affinity

Our findings consistently showed syndecan-1-dependent effects on collagen matrices indicating that this proteoglycan affects specific cell-matrix interactions to modulate its effect on cell migration. Syndecan-1 can associate with certain integrins [34], and we evaluated the β_1_ integrin subunit as it is common to all the fibrillar collagen binding integrins [35]. Deficiency of syndecan-1 did not affect the overall levels of β_1_ integrins (Figure 4D). However, the β_1_ integrin subunit can assume active and inactive conformations conferring dramatic differences in substrate affinity [36]. Using a conformation-specific antibody, we found active β_1_ present on the basolateral surface of B2b^shRNA.luc^ cells but largely absent in B2b^shRNA.Sdc1^ cells lacking syndecan-1 (Figure 4D). Because α_2_β_1_ is the primary collagen binding integrin in most epithelia including the lungs [37], these data suggest that syndecan-1 governs the activation state of this receptor.

We tested the effects of syndecan-1 on the α_2_β_1_ integrin with cell adhesion assays in the presence of functional activating and inhibiting antibodies (Figure 4E). In the presence of isotype antibody, we again showed differential binding of B2b^shRNA.luc^ and B2b^shRNA.Sdc1^ cells to collagen (100% vs 50.5±8.4%, respectively). Blocking antibodies against the β_1_ integrin subunit or specific to the α_2_β_1_ integrin abrogated binding of both B2b^shRNA.luc^ and B2b^shRNA.Sdc1^ cell adhesion to collagen (β_1_: 9.3±2.5% vs. 9.7±3.0%, respectively; α_2_β_1_: 24.4±12.0% vs. 7.9±3.1%, respectively). In contrast, whereas addition of a β_1_ activating antibody did not significantly affect B2b^shRNA.luc^ adhesion to collagen (87.0±15.7%), it did augment adhesion of syndecan-1-deficient B2b^shRNA.Sdc1^ cells compared to isotype control (87.9±11.0% vs. 50.5±8.4%, respectively). Forced β_1_ integrin subunit activation did not rescue cell adhesion in the presence of an α_2_β_1_ integrin inhibitor for either B2b^shRNA.luc^ or B2b^shRNA.Sdc1^ cells (30.9±15.5% vs. 17.5±10.5%, respectively). These data indicate that syndecan-1 modulates cell adhesion to collagen via the α_2_β_1_ integrin and suggests that this regulation occurs by controlling the affinity state of the integrin.

### Modulation of the α_2_β_1_ integrin during re-epithelialization

Because the presence of syndecan-1 appears to shift the α_2_β_1_ integrin to a higher affinity state, MMP7 shedding of syndecan-1 may release this control and deactivate the integrin to a lower affinity, thereby allowing more relaxed cell-matrix interactions that are permissive to cell migration. Indeed, whereas some degree of cell adhesion is needed to generate migratory traction, excessive adhesion impedes cell movement [38], [39]. In consideration of these concepts, our model would predict that in the presence of syndecan-1, the α_2_β_1_ integrin restrains cell migration during re-epithelialization. If true, forced activation of the β_1_ integrin subunit should slow re-epithelialization seen in *Sdc1^−/−^* ALI cultures. Moreover, inhibiting α_2_β_1_ integrin ligation should overcome the diminished migration seen in *Mmp7^−/−^* conditions where syndecan-1 persists at the cell surface.

**Figure 5:**
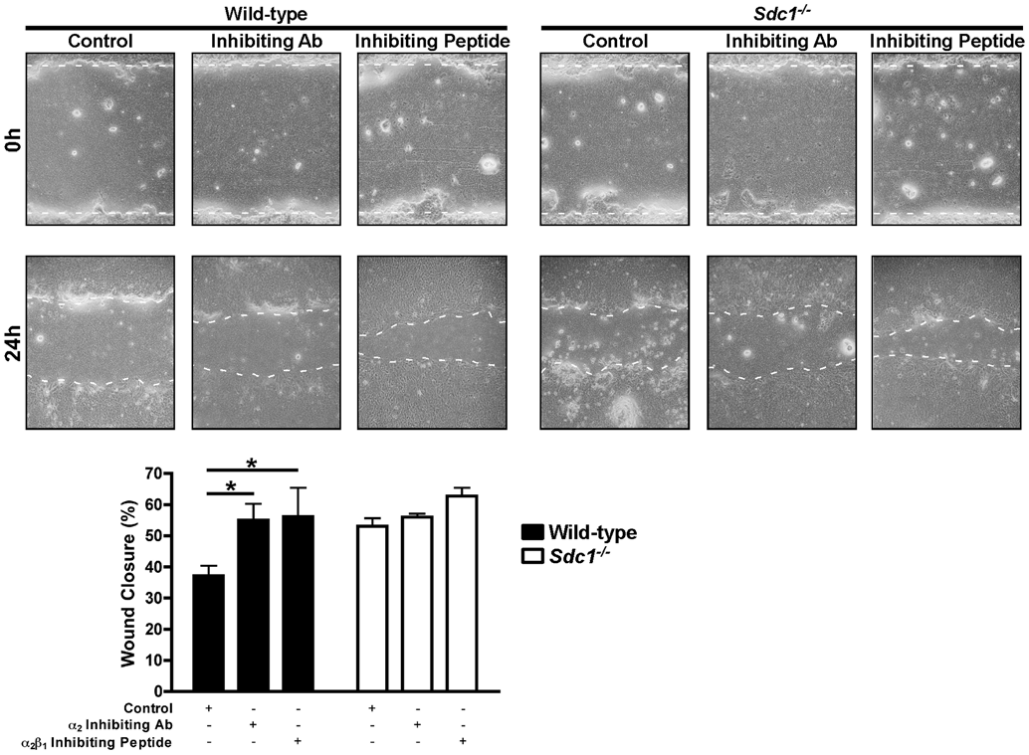
Wounded *Sdc1^−/−^* lung epithelium is unaffected byα_2_β_1_ integrin inhibition. Wild-type and *Sdc1^−/−^* ALI cultures were injured in the presence of a control (hamster isotype IgG_2_; 10 µg/ml), α_2_ integrin subunit inhibiting antibody (clone Ha1/29; 10 µg/ml) or α_2_β_1_ integrin inhibiting peptide (5 mM). The percent wound closure was determined 24 h after injury. *p<0.05 by 2-way ANOVA and Bonferroni analysis. n = 4; Original magnification×100.

**Figure 6:**
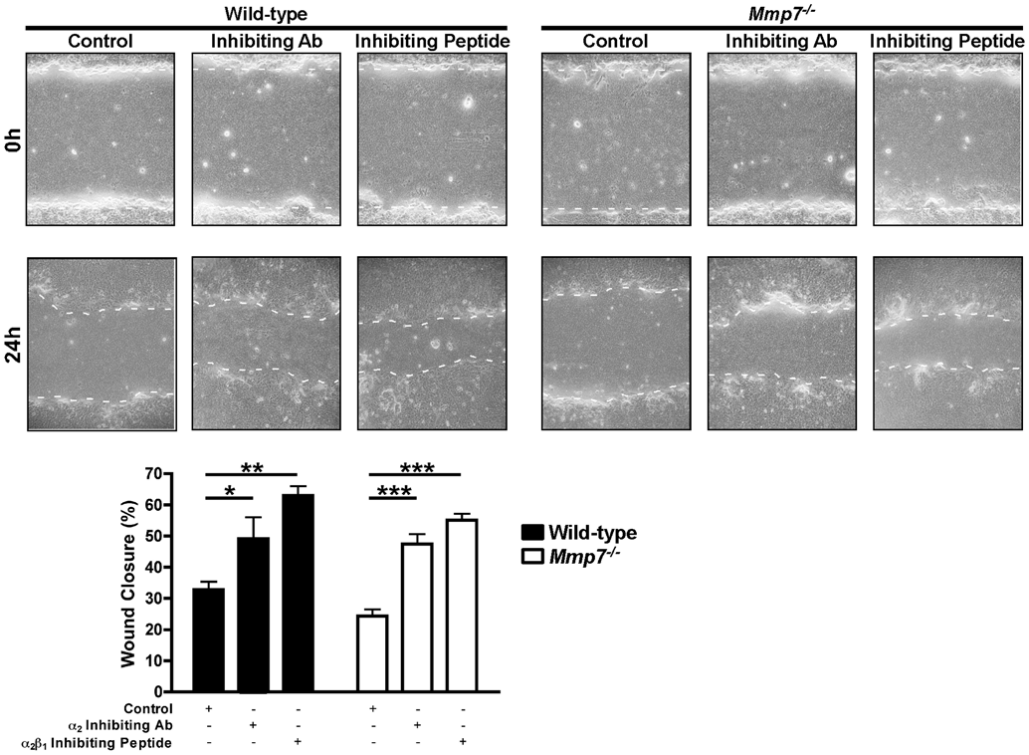
Wounded *Mmp7^−/−^* lung epithelium has augmented wound closure with α_2_β_1_ integrin inhibition. Wild-type and *Mmp7^−/−^* ALI cultures were injured in the presence of a control (hamster isotype IgG_2_; 10 µg/ml), α_2_ integrin subunit inhibiting antibody (clone Ha1/29; 10 µg/ml) or α_2_β_1_ integrin inhibiting peptide (5 mM). The percent wound closure was determined 24 h after injury. *p<0.05, **p<0.01, ***p<0.001 by 2-way ANOVA and Bonferroni analysis. n = 4; Original magnification×100.

To test these ideas, we first established that blocking the α_2_β_1_ integrin enhances wound repair in ALI airway epithelial cultures. Wounded WT ALI cultures had enhanced wound closure in the presence of either an α_2_ subunit inhibiting antibody or an α_2_β_1_ inhibiting peptide (Figure 5 and 6). In contrast, *Sdc1^−/−^* ALI cultures had no additional augmentation of wound closure suggesting the α_2_β_1_ integrin contributed minimally to cell migration in the absence of syndecan-1 (Figure 5). However, and consistent with our hypothesis, the α_2_ subunit and α_2_β_1_ integrin inhibitors both increased the wound closure rate of injured *Mmp7^−/−^* ALI cultures (Figure 6). These data support our idea that in the presence of syndecan-1, the higher affinity state of the α_2_β_1_ integrin restrains migration of the repairing airway epithelium.

**Figure 7:**
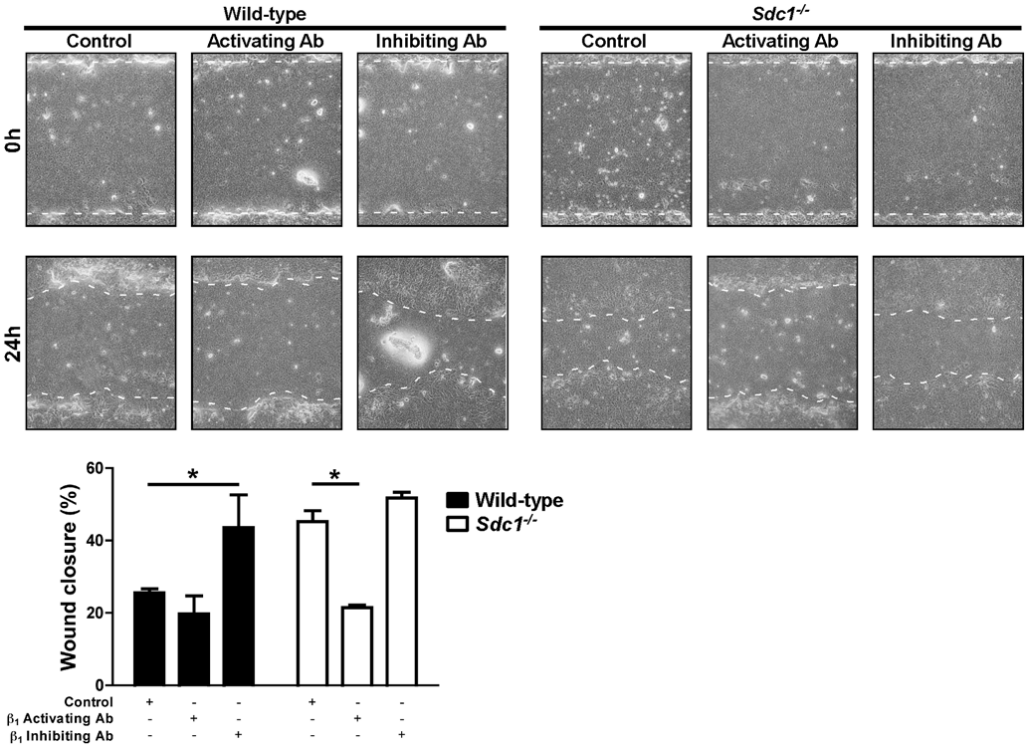
Wounded *Sdc1^−/−^* lung epithelium has attenuated repair with forced β_1_ integrin subunit activation. Wild-type and *Sdc1^−/−^* ALI cultures were injured in the presence of a control (rat isotype IgG, 1 µg/ml) or β_1_ integrin subunit activating (clone 9EG7, 1 µg/ml) or inhibiting (clone AIIB2, 1 µg/ml) antibodies. The percent wound closure was determined 24 h after injury. *p<0.05 by 2-way ANOVA and Bonferroni analysis. n = 4; Original magnification×100.

**Figure 8:**
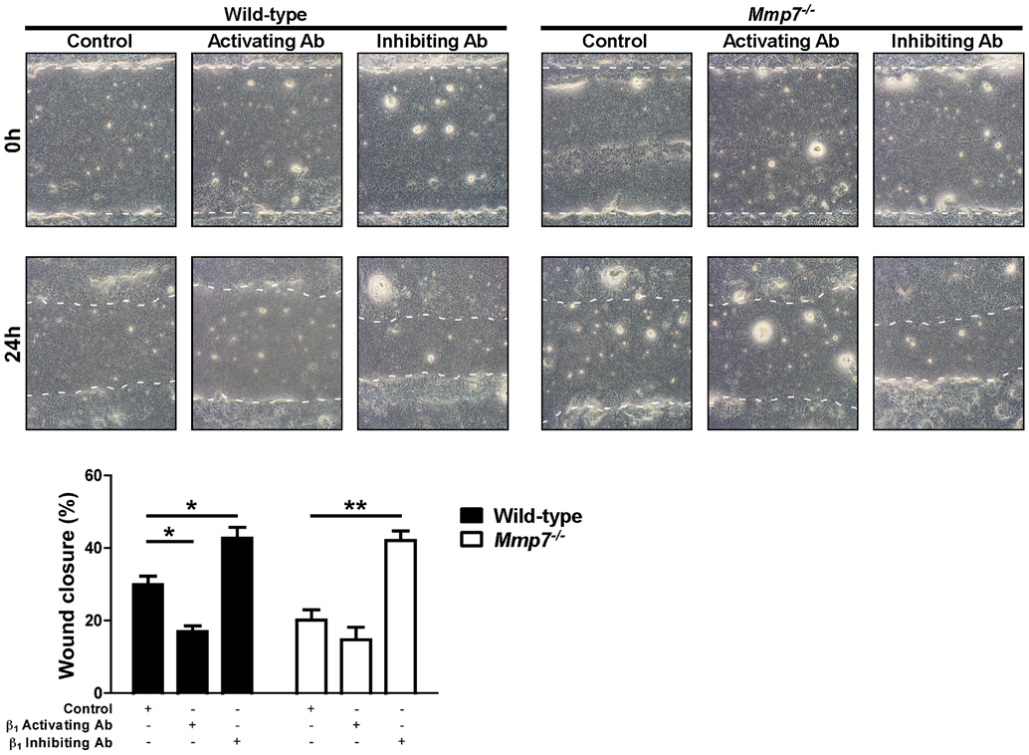
Wounded *Mmp7^−/−^* lung epithelium has enhanced repair with β_1_ integrin subunit inhibition. Wild-type and *Mmp7^−/−^* ALI cultures were injured in the presence of a control (rat isotype IgG, 10 µg/ml) or β_1_ integrin subunit activating (clone 9EG7, 10 µg/ml) or inhibiting (clone AIIB2, 1 µg/ml) antibodies. The percent wound closure was determined 24 h after injury. *p<0.01, **p<0.001 by 2-way ANOVA and Bonferroni analysis. n = 4; Original magnification×100.

Next, we wounded WT and *Sdc1^−/−^* ALI cultures in the presence of control, β_1_-activating, or β_1_-inhibiting antibodies (Figure 7). Inhibition of β_1_ integrins augmented wound closure rates of injured WT ALI cultures to levels equivalent to *Sdc1^−/−^* cultures. Conversely, forced activation of the β_1_ integrin subunit slowed the wound closure rate of injured *Sdc1^−/−^* ALI cultures to that of WT conditions. Congruous with our hypothesis, inhibiting β_1_ integrin subunit restored wound closure rate of *Mmp7^−/−^* cultures to WT levels (Figure 8). In WT ALI cultures, the activating β_1_ integrin antibody significantly slowed wound closure relative to control conditions, but this effect was seen at the higher concentration used (compare Figures 7 and 8). In contrast, the higher concentration of β_1_ integrin activating antibody had no effect on *Mmp7^−/−^* wound closure rate suggesting that the β_1_ integrin subunit was already maximally activated.

## Discussion

Injury opens avenues for pathogenic entry across a breached barrier. Therefore, the body has evolved mechanisms to recruit inflammatory cells to combat potential pathogens while quickly repairing the damaged tissue. The lungs, in particular, have adapted its mucosal surface to utilize MMP7 in regulating both inflammation and repair. Various injuries stimulate a rapid and dramatic induction of MMP7 production by the wounded epithelium [3]–[11]. This expression is necessary for recruiting inflammatory cells while promoting re-epithelialization [3]–[6]. Our group previously reported MMP7 governs the inflammatory response through the shedding of syndecan-1 [4]. Here, we provide evidence that syndecan-1 shedding also serves to promote re-epithelialization.

Damaged epithelium start spreading within minutes after injury while the initiation of cell migration is delayed by several hours [30], [31]. Our data show that MMP7 regulates the migration component of the re-epithelialization process. Migration is a complex process that is affected by multiple interactions between the cell and its environment [40]. In particular, cell-matrix interaction must be tightly controlled to ensure affinity is adequate for traction but not so excessive as to prevent forward migration [38], [39]. Therefore, MMP7 seems to be required for proteolysis of a substrate that normally restrains cell migration. MMP7 is the primary sheddase of syndecan-1 in the lungs after injury [4]. Our findings demonstrate that MMP7 shedding of syndecan-1 also facilitates wound closure. Indeed, syndecan-1 restricts migration as demonstrated with the augmented wound closure in conditions lacking syndecan-1. Moreover, lung epithelial cells transfected with syndecan-1 resistant to MMP7 proteolysis attenuates cell migration compared to wild-type syndecan-1. The *Sdc1^−/−^* and *Mmp7^−/−^* conditions also have opposite repair phenotypes, and these effects are consistent with the idea that MMP7 shedding of syndecan-1 releases restraints to cell migration.

Our data show that syndecan-1 augments cell adhesion to collagen. This increase in cell-matrix binding, in turn, promotes cell spreading but represses cell migration. Numerous studies have documented a functional coupling of syndecan-1 with integrins [34]. Our results describe how syndecan-1 indirectly governs cell-matrix binding in the lungs by modulating α_2_β_1_ integrin affinity. Syndecan-1 shedding could promote cell migration by re-organizing cell interactions with the environment [25], [40]. Indeed, the loss of syndecan-1 has been shown to induce changes in the cellular machinery that promote migration [18], [19]. Integrin-mediated attachments can also promote cell spreading [41], but the cell may also require syndecan-1 as an additional signal to re-organize the actin cytoskeleton and facilitate cell spreading [42]–[49]. Certainly, changes in cell-matrix interaction can switch the functional outcome of the cell [50], [51]. Our findings indicate that syndecan-1 regulates the affinity state of the α_2_β_1_ integrin subunit, which in turn appears to coordinate cell attachments to the matrix that are required for spreading and migration. This activation event can occur quickly through a conformational change without any need for protein expression as the required components are already in place.

Syndecan-1 being functionally coupled to integrins has been demonstrated in other models [34]. For example, syndecan-1 regulates the activation state of α_v_β_3_ integrin potentially as a way for breast carcinoma cells to acquire a more invasive phenotype [44], [45]. Additionally, syndecan-1 associates with and facilitates the activation of α_v_β_5_ integrin in a fibroblast cell line [47]. Similarly, syndecan-1 was found to directly interact with and modulate the affinity state of α_v_β_3_ and α_v_β_5_ integrins in endothelial cells to facilitate angiogenesis *in vivo* [52]. Studies with keratinocytes show a migratory defect when deficient in syndecan-1, possibly through alterations in laminin 332 binding integrins [17], [53]–[55]. In contrast, *Sdc1^−/−^* dermal fibroblasts have increased migration, increased β_1_ and α_v_ integrin subunit expression and augmentation of α_v_ integrin subunit activity compared to WT conditions [56]. Recently, syndecan-1 was found to physically interact with the β_1_ integrin subunit [57] and augment cell adhesion to type I collagen by cooperating with α_2_β_1_ integrin [58]. Conversely, other studies specifically evaluating syndecan-1 effects on the β_1_ integrin subunit found no difference in expression and activation [19], [43], [46]. Syndecan-1 appears to have multiple possible interactions with various integrins, depending on the cell system that is used. This likely represents the fact that different lineages of cells express different repertoires of surface and intracellular proteins from which syndecan-1 can associate.

Syndecan-1 also appears to function in a tissue specific manner. Skin and corneal epithelium deficient in syndecan-1 have defective re-epithelialization *in vivo* apparently due to attenuated proliferative and migratory responses [16], [17], [59]. Interestingly, syndecan-1 overexpression also inhibits skin repair *in vivo* possibly through inhibitory actions from the syndecan-1 ectodomain [14]. One explanation for the different effects of syndecan-1 is that the skin and cornea have a stratified epithelium that is structurally and functionally distinct from the simple epithelium in the lungs.

A recent study using A549 cells, a carcinoma-derived alveolar type II cell line, reported that knockdown of syndecan-1 expression slowed cell migration [60]. The findings we present here directly contradict the results in the A549 cell line. A549 cell lines are cancerous in origin, and thus, changes associated with the transformed phenotype may have altered the functions of syndecan-1 and the pathways controlling cell movement, such as that seen with breast cancer cells [44], [45]. In contrast, we used a non-cancerous cell line (BEAS-2b) and organotypic cultures derived from primary epithelial cells, as well as *in vivo* models. Further investigation would be needed to fully understand the fundamental reasons for the different results found in these studies.

Together, our data support the conclusion that MMP7 facilitates re-epithelialization through the shedding of syndecan-1, which then changes the activation state of the α_2_β_1_ integrin to reduce cell affinity to collagen and remove restrictions to migration. MMP7 is induced in the lungs by injury and sheds syndecan-1 as a protective mechanism to recruit neutrophils while promoting re-epithelialization. Because inflammation and repair are two interrelated phenomena, MMP7 shedding of syndecan-1 could be the result of evolution co-opting two important biological processes into one unified mechanism.

## Materials and Methods

### Ethics Statement

All animal procedures were approved by the Institutional Animal Care and Use Committee at the University of Washington and the National Institute of Health Guide for the Care and Use of Laboratory animals.

### Antibodies and Peptides

Clara cell specific protein (CCSP) was immunostained in mouse tissue with a rabbit polyclonal antibody (Upstate, Lake Placid, NY). Syndecan-1 antibodies were clone B-B4 for immunostaining on human samples (BD Biosciences, San Jose, CA) and clone 281.2 for mouse tissue (kindly provided by P.W. Park; Children's Hospital, Harvard Medical School, Boston, MA). Active conformation β_1_ integrin subunit was immunostained on human tissue with clone 12G10 (Millipore, Billerica, MA) as previously described [61]. All β_1_ integrin was immuostained with clone AIIB2 (Developmental Studies Hybridoma Bank, Iowa City, IA; Deposited by C. Damsky, UCSF). Appropriate Alexa Fluor labeled secondary antibodies were from Invitrogen (Carlsbad, CA).

Functional integrin activators and inhibitors were as follows. Clone BHA2.1 was used as a functional α_2_β_1_ integrin inhibitor in human cells [62], [63]. The α_2_β_1_ ligand peptide (Anaspec, Fremont, CA) inhibits integrin binding to collagen [64] and was used in mouse cells. Clone Ha1/29 (BD Biosciences) was used as a mouse α_2_ subunit inhibitor [65], [66]. Clones HUTS-21 [67] and 9EG7 [68], [69] were used respectively as human and mouse β_1_ subunit activating antibodies. Clone AIIB2 was used to inhibit β_1_ subunit function in both human and mouse systems [70]. Although the clone AIIB2 antibody was generated toward the human β_1_ subunit, cross-reactivity with several species have been established [71]–[73], and preliminary studies showed effect in blocking adhesion of murine cells (data not shown). All functional experiments were matched with isotype antibody controls from Santa Cruz Biotechnologies (general rat isotype), BD Biosciences (Armenian Hamster IgG_2_) and BioLegend (all mouse and rat monoclonal isotype controls). The one exception is when 9EG7 (rat IgG_2a_) and AIIB2 (rat IgG_1_) were both used (Figures 7 and 8), a general rat isotype IgG antibody (Santa Cruz Biotechnologies) was used as a common control. Preliminary experiments showed no difference between rat monoclonal IgG isotypes and the general rat IgG control (data not shown).

### Lung injury models

Air-liquid interface (ALI) cultures were created from wild-type (WT), MMP7-null (*Mmp7^−/−^*) and syndecan-1-null (*Sdc1^−/−^*) mice all on a C57BL/6 background, and wound closure assays were performed as previously described [6]. Additionally, time-lapse microscopy of the repairing ALI culture obtained DIC images every 6 min over 24 h on a DeltaVision Olympus IX71 inverted microscope using a 20x/0.75 U plan Apo objective and a Photometric Coolsnap HQ camera (Applied Precision, Issaquah, WA).

The naphthalene injury model was used to study repair *in vivo* [6]. WT, *Mmp7^−/−^* and *Sdc1^−/−^* mice had intraperitoneal injections of 200 mg/kg sterile naphthalene dissolved in corn oil. All mice were injected between 8–10 am to minimize diurnal variations in naphthalene metabolism. Mice were sacrificed at the defined time point, bronchoalveolar lavage fluid was collected and the lung tissue was processed for histology.

### Knockdown and Overexpression Cell Lines

The pSM2 vector containing shRNA specific for the human syndecan-1 gene or the luciferase gene was purchased from Open Biosystems (Huntsville, AL). Wild-type human MMP7 was mutated to contain a furin cleavage site at the junction of the pro- and catalytic domain (generously provided to us by D. Madtes; Fred Hutchinson Cancer Research Center, Seattle, WA). This construct was subcloned using *BamHI* and *NotI* restriction sites into the pBM-IRES-blastocidin retroviral expression plasmid (kindly provide by E. Raines; University of Washington, Seattle, WA). The full-length WT mouse syndecan-1 (mSdc1) was also PCR cloned from cDNA created from whole lung RNA, and the PCR product was inserted into the GFP Fusion TOPO TA expression kit (Invitrogen, Carlesbad, CA). A non-cleavable syndecan-1 (NC-mSdc1) was created by replacing the juxtamembrane MMP cleavage site with 15 amino acids from the human CD4 juxtamembrane sequence as previously described [25]–[27].

Stable knockdown cells were created using a retroviral transduction system. For both shRNA and overexpressing retroviral expression systems, retroviral particles were produced by transiently transfecting plasmid DNA into the PhiNx amphoteric packaging cell line (ATCC, Manassas, VA; Deposited by G. Nolan) [74]. Target cells were infected with retroviral conditioned medium containing 10 µg/ml DEAE-Dextran for 16 h at 33°C before being transferred to 37°C in fresh medium [75]. Two days after infection, medium containing the appropriate selection antibiotic was added and stably transduced cell lines were selected. Additionally, cells were transiently transfected to express mSdc1 or NC-mSdc1 with Lipofectamine 2000 (Invitrogen) following manufacturer's directions.

### Histology and Immunostaining

All immunostaining of ALI cultures and of cell lines were performed on 100% methanol-fixed cultures. H&E staining and CCSP immunofluorescent staining was performed on 10% formalin-fixed, paraffin-embedded naphthalene injured tissue sections as previously described [6]. Epifluorescence images were captured using an Olympus BX-51 fluorescence/DIC microscope with U plan Apo 40x/0.85 and 20x/0.70 objectives and an Olympus DP25 5.5 megapixel digital camera. All immunofluorescent slides were processed with identical conditions. Images were captured with equal exposures and microscope settings. When necessary, minor linear changes to intensity were made equally with ImageJ software.

### Migration, Spreading and Adhesion Assays

Assays to evaluate cell-matrix interactions effects on components of repair were employed. Cell spreading was evaluated as previously described [6]. Plated cells were allowed to spread for 60 min on various matrices, and the percent of cells spread compared to all plated cells was determined. We also evaluated cell migration using a modified colloid gold migration assay [33]. The protocol was modified so that experiments were performed on Permanox chamber slides (Nunc, Rochester, NY). In each experiment of the migration assay, at least 10 cells were randomly chosen, and the migration area was measured with ImageJ software. Scratch wound assays were also used as a method to measure migration and wound closure [5], [6].

Cell adhesion to a matrix was determined with a modified cell adhesion assay [76]. Cells were pre-labeled with 5 µM calcein AM (Alexis Biochemicals, San Diego, CA) in 10% FBS DMEM medium for 30 min. Cells were then washed and lifted from the culture plate by incubating in PBS-EDTA at 37°C for 10 min. All cells were collected and washed with PBS before plating onto matrix coated 96-well round-bottom plates in 1% FBS phenol-free DMEM (10,000 cells per well in 100 µl total volume). To synchronize the initial cell-matrix contact, plates were centrifuged at 30×g for 3 min. Total fluorescence (excitation 485, emission 530) was measured at 0 h as a baseline. After 30 min at 37°C, plates were washed with an electronic multichannel pipettor set at a consistent ejection force for all experiments to remove non-adherent cells. After three washes of PBS, 100 µl of 1% FBS phenol-free DMEM was replaced into each well and the overall fluorescence was measured. The percent adherence was determined by a ratio of the fluorescent signal at 30 min compared to baseline. When indicated, cell adhesion assays were performed in the presence of isotype and functional activating and inhibiting antibodies. To compare among different experiments, which may require different control antibodies, all data was normalized to the positive control condition and presented as a relative adhesion percent.

## Supporting Information

Video S1 Wound closure of wild-type and MMP7−/− ALI cultures. Wild-type and MMP7−/− ALI cultures were injured and wound closure was observed over 24 hours.
(21.22 MB MOV)
